# The NEtherlands Cervical Kinematics (NECK) Trial. Cost-effectiveness of anterior cervical discectomy with or without interbody fusion and arthroplasty in the treatment of cervical disc herniation; a double-blind randomised multicenter study

**DOI:** 10.1186/1471-2474-11-122

**Published:** 2010-06-16

**Authors:** Mark P Arts, Ronald Brand, Elske van den Akker, Bart W Koes, Wilco C Peul

**Affiliations:** 1 Department of Neurosurgery, Medical Center Haaglanden, The Hague, The Netherlands; 2 Department of Neurosurgery, Leiden University Medical Center, Leiden, The Netherlands; 3 Department of Medical Statistics & Bio Informatics, Leiden University Medical Center, Leiden, The Netherlands; 4 Department of Medical Decision Analysis, Leiden University Medical Center, Leiden, The Netherlands; 5 Department of General Practice, Erasmus Medical Center, Rotterdam, The Netherlands

## Abstract

**Background:**

Patients with cervical radicular syndrome due to disc herniation refractory to conservative treatment are offered surgical treatment. Anterior cervical discectomy is the standard procedure, often in combination with interbody fusion. Accelerated adjacent disc degeneration is a known entity on the long term. Recently, cervical disc prostheses are developed to maintain motion and possibly reduce the incidence of adjacent disc degeneration. A comparative cost-effectiveness study focused on adjacent segment degeneration and functional outcome has not been performed yet. We present the design of the NECK trial, a randomised study on cost-effectiveness of anterior cervical discectomy with or without interbody fusion and arthroplasty in patients with cervical disc herniation.

**Methods/Design:**

Patients (age 18-65 years) presenting with radicular signs due to single level cervical disc herniation lasting more than 8 weeks are included. Patients will be randomised into 3 groups: anterior discectomy only, anterior discectomy with interbody fusion, and anterior discectomy with disc prosthesis. The primary outcome measure is symptomatic adjacent disc degeneration at 2 and 5 years after surgery. Other outcome parameters will be the Neck Disability Index, perceived recovery, arm and neck pain, complications, re-operations, quality of life, job satisfaction, anxiety and depression assessment, medical consumption, absenteeism, and costs. The study is a randomised prospective multicenter trial, in which 3 surgical techniques are compared in a parallel group design. Patients and research nurses will be kept blinded of the allocated treatment for 2 years. The follow-up period is 5 years.

**Discussion:**

Currently, anterior cervical discectomy with fusion is the golden standard in the surgical treatment of cervical disc herniation. Whether additional interbody fusion or disc prothesis is necessary and cost-effective will be determined by this trial.

**Trial Registration:**

Netherlands Trial Register NTR1289

## Background

Anterior cervical discectomy (ACD) is the basic surgical treatment of patients with radicular pain caused by cervical disc herniation. In 1958, Cloward, Smith and Robinson first described anterior cervical decompression with the use of autologous iliac crest interbody graft (ACDF)[1]. Shortly after, Hirsch debated the necessity of interbody fusion[2]. The results of various prospective randomised trials suggest that interbody fusion may not be necessary in all cases, although due to methodological flaws no solid conclusions can be drawn[3-9]. The Cochrane Review even mentioned advantages of anterior discectomy only (e.g. costs, operation time and return to work)[10].

At present, ACDF is defined as the golden standard for cervical disc herniation to maintain disc height, cervical alignment, and promote bony fusion. However, arthrodesis of a motion segment will lead to increased degenerative changes at the adjacent level. The concept of accelerated adjacent disc degeneration (AADD) is widely discussed and Hilibrand et al. reported an annual incidence of 2.9% symptomatic AADD after fusion[11].

The main rationale of artificial disc replacement is motion preservation with subsequent prevention of adjacent disc degeneration. Various prospective randomised trials have shown that cervical disc prosthesis is a safe and reliable alternative to cervical fusion[12-17]. However, limited studies have focused on symptomatic AADD and the follow-up period is short[18,19].

In the NEtherlands Cervical Kinematics (NECK) trial, we will randomly and blindly compare anterior cervical discectomy *sec *(ACD), with anterior discectomy with fusion (ACDF), and anterior discectomy with disc prosthesis (ACDP) in 3 treatment groups. We hypothesise a difference in symptomatic adjacent level disease in favour of disc prosthesis and better clinical outcome and self-assessment measured by the Neck Disability Index (NDI). As such we will evaluate the clinical appropriateness and superiority of disc prosthesis on one hand, cervical fusion on the other hand, and compare this to discectomy without any implant. Moreover, we will identify possible subgroups of patients who will substantially benefit from one of the allocated surgical treatments.

## Methods/Design

We designed an observer and patient blinded randomised cost-effectiveness trial in the treatment of cervical disc herniation in which three surgical techniques are compared in a parallel group design. Adjacent level disease will be used as primary outcome measure and NDI as secondary outcome. As such we will evaluate the clinical appropriateness and superiority of disc prosthesis on one hand, cervical fusion on the other hand, and compare this to discectomy without any implant. The follow-up period will last 5 years.

In order to collect enough patients, a multicenter design is necessary. The clinical centers that are planned to recruit patients into the NECK trial are: Medical Center Haaglanden, The Hague; Leiden University Medical Center; University Medical Center Groningen; Medical Center Alkmaar; and University Medical Center Maastricht. The protocol was approved by the ethics committee/IRB in each participating hospital.

The primary question of the NECK trial is whether ACDP has a lower incidence of symptomatic adjacent disc degeneration compared to ACD and ACDF. Symptomatic adjacent level disease is defined as development of new symptoms (i.e. neck pain, radiculopathy, or myelopathy) referable to a motion segment adjacent to the site of the previous anterior surgery. In addition, we want to identify certain subgroups that may benefit more of one of the allocated treatments.

### Patients

All patients between 18 and 65 years with monoradicular symptoms in one or both arms lasting more than 8 weeks are eligible for the NECK trial. MRI must confirm cervical disc herniation and/or osteophyte in accordance with clinical symptoms. Additional inclusion and exclusion criteria are listed in Table 1.

**Table 1:** Selection criteria for trial eligibility.

**Inclusion criteria**
Age 18 - 65 years
Radicular signs and symptoms in one or both arms
At least 8 weeks prior conservative treatment
Radiographic diagnosis of cervical disc herniation and/or osteophyte at 1 level (C3-C4 to C7-T1) in accordance with clinical signs and symptoms
Informed consent
**Exclusion criteria**
Previous cervical surgery (either anterior or posterior)
No motion of the index level on dynamic studies
Increased antero-posterior motion of the index level on dynamic studies (>3 mm)
Involved disc level fused or very narrow (central <3 mm)
Severe segmental kyphosis of involved disc level (>3 degrees)
Neck pain only
Symptoms and signs of chronic myelopathy
Infection, bone disease, neoplasm or trauma of the cervical spine
Spinal anomaly (Klippel-Feil, Bechterew, OPLL)
Severe mental or psychiatric disorder
Inadequate Dutch language
Planned (e)migration abroad in the year after inclusion

Patients are referred by a neurologist with MRI of the cervical spine. During the first visit to the neurosurgical outpatient clinic, the patient's history and a standard neurological examination will be documented. Conform our selection criteria, the neurosurgeon decides whether a patient is eligible for the NECK trial. The study will be explained to the patient and, in case of a positive reaction, an appointment is made with one of the research nurses. Because the patient needs some time to consider participation, the first visit to the research nurse is planned after at least 2 days. After informed consent, the questionnaires, outcome measures, and baseline variables are recorded.

### Randomisation procedure

Patients will be randomly allocated to ACD, ACDF, or ACDP. Randomisation will take place on the operating room within 6 weeks after the first visit to the research nurse. A randomisation list is prepared for every participating hospital-nurse combination. Variable sized blocks of random numbers are formed to ensure equal distribution of the randomisation treatments over hospitals and research nurses. The data manager, who is not involved in the selection and allocation of patients, will prepare coded, sealed envelopes containing the treatment allocation. In the operating room, after induction of anaesthesia, the surgeon will open the envelope and the allocated treatment will be performed. Patients and research nurses will be kept blinded for the allocated treatment for 2 years.

### Intervention

Patients will be randomised into anterior discectomy *sec *(group A), anterior discectomy with interbody fusion (group B), and anterior discectomy with arthroplasty (group C). The participating surgeons have large experience in all three techniques. A standardized Case Record Form (CRF) will register the surgeon's findings and this CRF, together with the randomisation envelope, will be returned to the data center in a sealed envelope.

### (A) Anterior cervical discectomy (ACD)

All patients will be positioned supine with their neck in neutral position or slightly extended under general anaesthesia. The affected cervical disc level will be verified with fluoroscopy. A small transverse incision will be made either on the right side or the left side depending on the surgeon's preference. Medial to the carotic sheath, the pre-vertebral space will be opened and the anterior cervical spine will be exposed. Two distraction pins and the Caspar spreader will be placed in the affected segment. A standard anterior discectomy with the aid of microscope or loupe/headlight magnification (depending on the surgeon's preference) will be performed in all cases. The posterior longitudinal ligament will be opened and the nerve root and dura will be decompressed adequately. If required a vacuum drain will be placed and the wound will be closed in layers.

### (B) Anterior cervical discectomy with interbody fusion (ACDF)

Once the anterior discectomy has been performed, an interbody cage filled with bone substitute will be placed within the intervertebral space under fluoroscopic guidance. The type of cage depends on the surgeon's preference and daily practice. To prevent pain from the iliac crest, no autologous bone will be used.

### (C) Anterior cervical discectomy with disc prosthesis (ACDP)

The device used in the present trial is the Activ-C Flat artificial disc (B. Braun Aesculap, Germany)[20]. After the standard anterior discectomy is performed, the implant size will be determined and the endplates will be prepared for proper fitting of the prosthesis. The device will be inserted under slight distraction and fluoroscopic guidance. Whenever, for surgical-technical reasons, it is not possible to implant the disc prosthesis, an interbody fusion will be performed. Whenever this is impossible as well, only discectomy will be performed.

All patients will be encouraged to mobilise as soon as possible without a collar. Hospital admission will be 1-3 days, depending on the usual care of the participating hospital. Patients are stimulated to resume home activities and work as soon as possible.

### Baseline data

Baseline assessment includes demographics, hobbies, sports, work status, smoking status, neck and arm pain history of patient and family, medical history and co-morbidity, body mass index, and neurological signs and symptoms. The patient's satisfaction at work will be registered. The treatment preference of patient, surgeon and research nurse will also be assessed.

### Outcome assessment

We will assess the below described validated outcome parameters. Patients will not be informed about their previous scores. Follow-up examination will take place at 8, 26, 52, 104 and 260 weeks after randomisation. Patients will be neurologically examined and questionnaires will be filled in. At 2, 4, 12, 156 and 208 weeks after randomisation, questionnaires will be sent by mail (Table 2).

**Table 2:** Data collection and outcome measures.

	Intake surgeon	Intake research nurse	Informed consent	Surgery	Follow-up 2 weeks	Follow-up 4 weeks	Follow-up 8 weeks	Follow-up 12 weeks	Follow-up 26 weeks	Follow-up 52 weeks	Follow-up 104 weeks	Follow-up 156 weeks	Follow-up 208 weeks	Follow-up 260 weeks
Visit	1	2	3	4			5		6	7	8			9
Inclusion/exclusion criteria	X													
Informed consent			X											
Treatment preference	X	X												
Expected recovery	X	X												
Demographics & diagnosis	X	X												
Basic physical examination	X													
Neurological examination	X	X					X			X	X	X	X	X
Randomisation				X										
Dynamic X-ray	X						X			X	X	X	X	X
MRI	X										X			X
CT	X										X			X
NDI		X			X	X	X	X	X	X	X	X	X	X
McGill		X			X	X	X	X	X	X	X	X	X	X
VAS arm and neck		X			X	X	X	X	X	X	X	X	X	X
SF-36		X			X	X	X	X	X	X	X	X	X	X
HADS		X					X		X	X	X	X	X	X
Karasek		X			X	X	X	X	X	X	X	X	X	X
Likert					X	X	X	X	X	X	X	X	X	X
Macnab							X		X	X	X			X
EuroQol and VAS		X			X	X	X	X	X	X	X	X	X	X
Cost diaries								X	X	X	X	X	X	X
Complications				X			X			X	X	X	X	X
Re-operation							X			X	X	X	X	X

### Primary outcome measure

*Accelerated adjacent disc degeneration (AADD). *Although the evaluation of cervical disc degeneration is very important in this clinical setting, there is no established standardized nomenclature. Radiographic degenerative changes at the superior and inferior adjacent levels will be recorded by means of X-ray at intake and each follow-up moment. At 2 and 5 years follow-up both CT and MRI of the cervical spine will be performed. In this trial we will use a recently developed MRI-based grading system for cervical intervertebral disc degeneration, which has been shown reliable[21](Table 3) It is of uppermost importance that radiological adjacent level disease will be accompanied by concomitant complaints, but we will evaluate radiographic adjacent level disease without symptoms as well.

**Table 3:** MRI-based grading system for cervical intervertebral disc degeneration.

Grade	Nucleus signal intensity	Nucleus structure	Distinction of nucleus and annulus	Disc height
I	Hyperintense	Homogenous, white	Clear	Normal
II	Hyperintense	Inhomogenous with horizontal band, white	Clear	Normal
III	Intermediate	Inhomogenous, grey to black	Unclear	Normal to decreased
IV	Hypointense	Inhomogenous, grey to black	Lost	Normal to decreased
V	Hypointense	Inhomogenous, grey to black	Lost	Collapsed

### Secondary outcome measures

### 1) Neck Disability Index (NDI)

The NDI is a 10-item questionnaire on 3 different aspects; pain intensity, daily work related activities and non-work related activities. Each item is scored from 0 to 5 and the total score ranges from 0 (best score) to 50 (worst score). The NDI is a modification of the Oswestry Low Back Pain Index and has been shown to be reliable and valid for patients with cervical pathology[22-24].

### 2) Short-Form 36 (SF-36)

This is a generic health status questionnaire that can easily be filled out at home. The questionnaire consists of 36 items on physical and social status of the patient subdivided in 8 domains; 1) physical functioning, 2) physical restrictions, 3) emotional restrictions, 4) social functioning, 5) somatic pain, 6) general mental health, 7) vitality and 8) general health perception. The questions are scored on a scale of 0 (worst health) to 100 (ideal health). This questionnaire has been used frequently and is validated in surgical studies on spinal column pathology[25-27].

### 3) Pain

Pain intensity, measured by Visual Analogue Score (VAS) of arm and neck and McGill pain Questionnaire is chosen as secondary outcome measure.

#### VAS arm pain

This parameter will measure the experienced pain intensity in the arm during the week before visiting the research nurse. Pain will be assessed on a horizontal 100 mm scale varying from 0 mm (no pain) to 100 mm (worst pain imaginable). Patients do not see the results of earlier assessments and will score the pain experienced at the visit. Reliability, validity and responsiveness of VAS have been shown[28].

#### VAS neck pain

Since many patients with radicular arm pain have neck pain as well, we will also measure the intensity of neck pain.

#### McGill Pain Questionnaire

The McGill Pain score consists of 4 parts: 1) quality and intensity of pain, 2) effects of pain on daily activities, 3) VAS, and 4) distribution and course of pain. The McGill pain score is shown to be highly effective to evaluate the effects of treatment on pain[29]. The Dutch version of McGill pain score will be used in this study[30].

### 4) Karasek Job Content Questionnaire

The Job Content Questionnaire is developed by Karasek to measure the on-the-job impact of chronic health problems and/or treatment[31]. We will use the Dutch version of the Karasek which has been shown reliability and validity[32].

### 5) Hospital Anxiety Depression Scale (HADS)

The HADS is a self-assessment scale which is developed to detect anxiety and depression in patients attending a medical clinician and has been shown reliable and valid[33]. The HADS consists of a 7-item depression scale and a 7-item anxiety scale. The score ranges from 0-21 with a high score being indicative for depression/anxiety.

### 7) Perceived recovery

This is a 7-point Likert scale measuring the perceived recovery, varying from "complete recovery" to "worse than ever". This outcome scale has been used in previous studies and is regarded valid and responsive to change[34]. Complete recovery and almost complete recovery are defined as good result. Next to this global self-assessment, a job and hobby specific Likert will be scored. During the intake of the study the patient will be asked to rank their 5 most important functional disabilities in daily life, which they can use in their own evaluation overall and in separate items. Moreover, the expected recovery from both patient, surgeon and research nurse will be evaluated.

### 8) Utility measurements

The EuroQol (EQ-5D) measures 5 dimensions (mobility, self-care, daily activities, pain/discomfort, anxiety/depression), on a 3 point scale (no, some, or extreme problems). For each health state described by the patients, a utility score can be calculated that reflects society's valuation of that health state. The Dutch tariff for the EQ-5D will be used[35]. Similarly, SF-6D utilities will be calculated from the SF-36 profiles[36]. Whereas the EQ-5D and SF-6D provide society's assessment of the patients' health, the patients themselves will also assess their own health on VAS, ranging from 0.0 (as bad as death) to 1.0 (optimal health). Both the EQ-5D and the VAS will be reported in questionnaires filled out at home.

### Other outcome measures

### 1) Costs

The direct medical costs of hospital admission (fixed costs per admission, and variable costs per admission day) and surgery (including personnel and implants) will be estimated in all participating hospitals for cost-analysis. Using cost diaries, the patients will register other medical care (including physiotherapy, visits to general practitioners and medical specialists, nursing care, and medication) and non-medical costs (including out-of-pocket expenses, domestic help, and absenteeism). Each diary will cover 3 months and the research nurse will go through the diary with the patient on every follow-up visit, throughout the study period of 2 years. Costs will be calculated using standard prices, including time and travel costs [37].

To estimate the indirect costs, like productivity costs, patients will register absenteeism in the diary and the research nurse will register the patient's work situation, work efficiency, and gross income on follow-up moments. Absenteeism will be valued to the friction-cost method.

### 2) Incidence of re-operations

In previous studies concerning spine surgery the incidence of re-operations has been used to assess outcome. Also in this study, we will assess the incidence of re-operation. Every surgical re-exploration in both groups will be considered as re-operation.

### 3) Complications

A systematic assessment of complications (including wound infection, deep venous thrombosis, urine tract infection, haematoma, progressive neurological deficit, dysphagia, and hoarseness) will be carried out by the surgeon and research nurse. Surgeons will also document perioperative complications like cerebrospinal fluid leakage, vascular injury, nerve root damage, and malposition of the implant.

### 4) Radiographic evaluation

Besides adjacent disc degeneration (primary outcome measure), we will also evaluate the cervical curvature, segmental motion, displacement or migration of the implant, and heterotopic ossification using the classification of McAfee[38].

### Sample size

The sample size calculation is based on the hypothesis that the incidence of symptomatic adjacent disc disease after cervical disc prosthesis is equal to anterior discectomy with or without interbody fusion. Based on the literature, the annual incidence of symptomatic adjacent disc disease after ACDF and ACDP is 7% and 0.65% respectively[18]. However, in that trial only 74 patients underwent disc arthroplasty and the probability of adjacent disc degeneration may be higher. Therefore, in our study, we assume an annual incidence of adjacent disc degeneration of 2% after arthroplasty and 7% after interbody fusion for the power calculations. We require a power of 90%, a significance level of 0.05, and assume committed accrual duration of 3 years. We plan a group sequential design with 2 interim analyses using a survival model approach for "time-to-degeneration" and a test-for-superiority approach. The East software (version 5) is used for the design of this study. The assumptions (2% vs. 7% to be detected) correspond to a Hazard Ratio of 0.28. With an accrual of 150 patients per year during 3 years, the required power is attained for a two-group comparison. Since we intend to perform two 2-group comparisons separately, we need 1.5*450 = 675 patients in total. Assuming a loss to follow-up of 10%, a total of 750 patients are needed. The interim analyses are performed only for the main comparison of ACDF versus ACDP. Stopping is allowed for futility as well as efficacy and follows prespecified alpha and beta spending rules.

### Data Analysis

The data will be analysed according to the stopping rules, i.e. after approximately 1.8, 2.6 and 3.2 years under H0 and 2.2, 3.2 and 4.2 years under H1. The actual analysis times depend on the number of events observed (15, 30 and 45). This establishes an answer to the main objectives in terms of the comparison of ADCF to ADCP. Long-term analyses of all results will be conducted irrespective of an early achievement of futility or efficacy establishing a comparison at 52, 104, 156, 208 and 260 weeks. However, these analyses will be performed after the final analysis of the trial has reached the conclusion of efficacy or futility. For all analyses, assessments performed at the first outpatient visit will be taken as baseline. Baseline comparability will be analysed by descriptive statistics to determine whether randomisation was successful. Baseline measurements will be used as covariates in the analyses to increase power. Differences in outcome measures between the 2 pairs of groups, together with 95% confidence intervals, will be calculated. All data are analysed according the "intent-to-treat principle".

The primary research objective will be tested using two likelihood ratio tests for the hazard ratio's, one for the comparison to ACD and one to ACDF. In case of homogeneity of effects, a pooled effect (adjusting for interbody fusion) will be reported. Otherwise the two analyses will be reported separately. The secondary outcome continuous measures will be compared using two t-tests in the same way (or non-parametric counterparts in case of non-symmetrical or distributions with outliers), possibly after transformations of skewed distributions.

On the other hand, repeated measurements analyses of variance for the secondary outcome measures (the continuous outcome scales) will also be performed in order to compare the evolving patterns over time.

In addition, an explorative subgroup analysis is conducted to investigate whether the treatment effect varies over specific subgroups of patients (Table 4).

**Table 4:** Selected prognostic variables for subgroup analysis.

**Demographic variables**
Age ≤ 40 years versus > 40 years
Women versus men
High education versus low education
**Anamnestic and neurological variables**
Neck pain versus no neck pain
Quetelet index ≤ 30 versus > 30
**Radiological variables**
Uncovertebral osteophytes versus no osteophytes
Straight neck versus lordotic neck
Low disc (≤5 mm) versus high disc (>5 mm)

Data will be stored via the internet-based secure data management system "ProMISe" of the department of Medical Statistics and Bio Informatics[39]. The analyses will be carried out using appropriate statistical software (e.g. SPSS).

## Discussion

Since the introduction of anterior approach of the cervical spine by Cloward, Robinson and Smith[1], a dispute has started about the best surgical treatment. The purpose of all surgical procedures is removal of the intervertebral disc in order to decompress the nerve root and alleviate radicular pain. However, cervical instability and segmental collapse with recurrent radicular pain has been documented after anterior discectomy. For this reason, most surgeons in general hospitals perform anterior discectomy with interbody fusion while most academic surgeons perform a discectomy *sec *as a result of lack of evidence. The results of various randomised trials suggest that interbody fusion may not be necessary in all cases[3-9]. In fact, The Cochrane Review even mentioned advantages of anterior discectomy only; lower costs, shorter operation time and faster return to work[10].

One of the main drawbacks of arthrodesis of a motion segment is increased load and stress at the levels adjacent to the fusion site. The concept of accelerated adjacent disc degeneration (AADD) is widely discussed. Hilibrand et al. reported a large retrospective study of patients who underwent anterior discectomy with fusion. Symptomatic adjacent level degeneration occurred at a relative constant incidence of 2.9% annually[11]. However, data on asymptomatic patients suggest that intervertebral disc degeneration is a physiological process, which may be accelerated by interbody fusion[40,41].

The rationale of artificial disc replacement, mainly industry driven, is motion preservation with subsequent prevention of adjacent disc degeneration. Various prospective randomised trials have shown that cervical disc prosthesis is a safe and reliable alternative to cervical fusion[12-17]. Data focused on adjacent disc degeneration after arthroplasty is limited, but the incidence of AADD seems low[18,19]. In the largest randomised trial on cervical disc prosthesis by Mummaneni et al., radiographic evaluation of adjacent level degeneration was not assessed[16]. Moreover, the follow-up period in all conducted studies is too short for proper evaluation of AADD.

The primary concern on studies focused on AADD, is the low incidence of events. As outlined in our sample size calculation, 750 patients will need to be randomised to elucidate the dispute on prevention of AADD in disc arthroplasty studies. In addition, an extra treatment arm of anterior discectomy without fusion will be needed to justify the high costs of cages or disc prostheses. Bartels et al. have published a similar protocol on cervical disc herniation in which 3 surgical strategies are compared[42]. However, the NDI was taken as primary outcome measure. Therefore, in our opinion, the number of patients enrolled is not enough for scientific proof of possible reduced incidence of AADD after disc arthroplasty.

The NECK trial is designed to demonstrate the effectiveness and security of cervical disc prosthesis, focused on adjacent segment degeneration and functional outcome. ACDP will be randomly and blindly compared with ACD and ACDF. The only way to elucidate the dispute on AADD prevention, is a 3 arm randomised trial with large number of patients to be included. As such we will evaluate the clinical appropriateness and superiority of disc prosthesis on one hand, cervical fusion on the other hand, and compare this to discectomy without any implant.

## Abbreviations

NECK: netherlands cervical kinematics; ACD: anterior cervical discectomy; ACDF: anterior cervical discectomy with fusion; ACDP: anterior cervical discectomy with prosthesis; NDI: neck disability index; VAS: visual analogue scale; AADD: accelerated adjacent disc degeneration; MRI: magnetic resonance imaging; CT: computed tomography; HADS: hospital anxiety depression scale.

## Competing interests

The authors declare that they have no competing interests.

## Authors' contributions

MA designed the protocol, is primary investigator and coordinator of the trial. RB has contributed to the case record forms, is responsible for the sample size calculation and implementation of the trial data management using the ProMISe software. EA is responsible for the design of the cost-effectiveness analysis. WP is the principal investigator, responsible budget holder and supervisor. All authors participated in the trial design and coordination. All authors read and approved the final manuscript.

## Pre-publication history

The pre-publication history for this paper can be accessed here:

http://www.biomedcentral.com/1471-2474/11/122/prepub
